# Reinvigorating Modern Breadmaking Based on Ancient Practices and Plant Ingredients, with Implementation of a Physicochemical Approach

**DOI:** 10.3390/foods10040789

**Published:** 2021-04-07

**Authors:** Vasileia Sereti, Athina Lazaridou, Costas G. Biliaderis, Soultana Maria Valamoti

**Affiliations:** 1 Laboratory of Food Chemistry and Biochemistry, Department of Food Science and Technology, School of Agriculture, Aristotle University of Thessaloniki, P.O. Box 235, 54124 Thessaloniki, Greece; vasisere@agro.auth.gr (V.S.); biliader@agro.auth.gr (C.G.B.); 2 Laboratory for Interdisciplinary Research in Archaeology (LIRA), Department of Archaeology, School of History and Archaeology, Aristotle University of Thessaloniki, 54124 Thessaloniki, Greece; sval@hist.auth.gr; 3 Center for Interdisciplinary Research and Innovation, Aristotle University of Thessaloniki (CIRI-AUTH), Balkan Center, Buildings A & B, 10th km Thessaloniki-Thermi Rd, P.O. Box 8318, 57001 Thessaloniki, Greece

**Keywords:** prehistoric grinding practices, ancient grain flours, breadmaking, starch gelatinization, dough rheology, bread quality parameters

## Abstract

In this study, the potential use of ancient plant ingredients in emerging bakery products based on possible prehistoric and/or ancient practices of grinding and breadmaking was explored. Various ancient grains, nuts and seeds (einkorn wheat, barley, acorn, lentil, poppy seeds, linseed) were ground using prehistoric grinding tool replicas. Barley-based sourdough prepared by multiple back-slopping steps was added to dough made from einkorn alone or mixed with the above ingredients (20% level) or commercial flours alone (common wheat, spelt, barley). Sieving analysis showed that 40% of the einkorn flour particles were >400 μm, whereas commercial barley and common wheat flours were finer. Differential scanning calorimetry revealed that lentil flour exhibited higher melting peak temperature and lower apparent enthalpy of starch gelatinization. Among all bread formulations tested, barley dough exhibited the highest elastic modulus and complex viscosity, as determined by dynamic rheometry; einkorn breads fortified with linseed and barley had the softest and hardest crust, respectively, as indicated by texture analysis; and common wheat gave the highest loaf-specific volume. Barley sourdough inclusion into einkorn dough did not affect the extent of starch retrogradation in the baked product. Generally, incorporation of ancient plant ingredients into contemporary bread formulations seems to be feasible.

## 1. Introduction

Recent archaeological research has revealed a wide range of plant materials, preserved mainly as charred plant remains, which have been most likely used for preparation of human food [1,2,3,4,5,6]. In certain cases, actual food remains from prehistoric Europe have been conserved through charring among the burnt debris in cooking areas or houses destroyed by fire [4]. The study of these findings, together with a good knowledge of food preparation techniques, as reflected in the remains of cooking installations, pots and grinding equipment found at prehistoric sites, allows glimpses into past culinary practices and the nutritional benefits of specific ways of food preparation; moreover, such remains allow for the exploration of an evolutionary continuum in the use of plant foods from prehistoric times to the present.

The archaeobotanical record reveals that a wide range of cereals and pulses constituted the staple foods of prehistoric European communities [7,8]. In the Neolithic and Bronze Age of Greece, the glume wheats hold a dominant position among the cereal species presumably used for food and included three species: einkorn, emmer and ‘new glume’ wheat (*Triticum. monococcum, T. dicoccum* and *T. timopheevi*, respectively). During the Bronze Age, spelt wheat (*T. spelta*) also appears for the first time at the end of the 3rd millennium B.C., while the free-threshing wheats (common wheat, *Triticum aestivum* and durum wheat, *Triticum durum*) had a more limited occurrence in prehistoric times [9]. Nowadays, the glume wheats, together with certain pseudocereal species, are widely referred to as ‘ancient grains’, whereas those described as ‘modern’ (naked) wheats (*T. aestivum* and *T. durum*) have already been grown since the Neolithic period. These free-threshing cereals, over time, became the dominant cereals grown for food in Western Asia and Europe at the expense of the glume wheats, perhaps because the latter have a lower productivity per cultivated area, a higher bulk volume when stored in their glumes and a greater labour input required for dehusking before milling [10,11]. An additional factor may have been the low quality of their storage proteins (i.e., inferior gluten aggregation properties and inadequacy to form a strong hydrated network structure), attributed to the low amount of high molecular weight polymeric gluten fractions, resulting in difficulties in dough handling and poor breadmaking performance [12]. Apart from the above wheat species, several other plant-derived materials could have been used for food preparation in prehistoric times, i.e., barley (*Hordeum vulgare*) grain and lentil (*Lens culinaris*) seeds, as well as seed of acorn nut (*Quercus* sp.), linseed (*Linum usitatissimum*) and poppy seed (*Papaver somniferum*) transformed into flour by grinding [4,6].

The transformation of cereals and other plant-derived food materials would have taken place with the facilities available at the time, which included stone grinding and pounding implements [13], cooking installations and pots [14]. The limited availability of remains of actual plant foods preserved in the archaeological record include different types of processed cereals, such as porridges and breads, although a clear distinction between these preparations is not straightforward [9] unless complete loaves or large bread fragments were sometimes preserved, presumably in the context of specific ritual or funerary events [3,15,16]. Precooked ground cereal foods reported in the archaeobotanical record have been identified from the end of the 3rd millennium B.C. in Mesimeriani Toumba, and they could correspond to a prehistoric bulgur or trachanas [4,17]. These actual food remains from a distant past provide a glimpse into prehistoric culinary practices, yet the recipes that led to such food remains are poorly understood; therefore, an attempt is being made to systematically investigate these aspects under the project of PlantCult [18].

Consumer interest in ancient wheat species (e.g., spelt wheat, einkorn wheat) with regard to their use in bakery products has recently emerged, particularly because these grains are rich sources of bioactive components and hence suitable for producing high value food products with enhanced nutrient content and health benefits, especially when used in the form of whole flours [19,20,21]. For instance, einkorn wheat seems to have nutritional properties that could distinguish it from common wheat varieties, although its rheological dough behavior may be inferior for breadmaking. Actually, einkorn wheat has a high content of ash, protein and essential amino acids, as well as various phenolic antioxidants [22,23,24]. Furthermore, another ancient cereal grain, barley, is an important source of cereal β-glucans, which are soluble dietary fibers, well-known for their hypocholesterolemic and hypoglycemic action [25]; in fact, barley flour is the main ingredient of several traditional bakery products made on the Greek island of Crete, such as barley rusks (‘Dakos’), that exist as staple food of the Mediterranean diet [26,27]. Other non-cereal raw materials, such as seeds from legumes, have been recently used to fortify breads due to their high content in carbohydrates, dietary fibers, vitamins, minerals, phytochemicals, and particularly proteins with a better amino-acids profile, ensuring a balanced diet when consumed in combination with cereals [28]. Additionally, seeds from nuts, such as acorns, have been incorporated into breads, mostly because of their antioxidant potential [29]. Oilseeds, such as linseed and their fractions, have been also engaged in modern breadmaking practices for nutritional enhancement of bakery products with soluble dietary fibers, essential amino acids and essential fatty acids, with the aim of reducing starch digestibility rates and improving atherogenic risk factors [30,31].

In this context, fortification of bakery items with ancient grains and seeds could have an impact to the wellbeing of individuals and contribute to the reduction of risk factors and/or management of chronic diseases, such as diabetes type 2 and cardiovascular diseases. Furthermore, adopting ancient breadmaking practices could give rise to new bakery products with improved nutritional attributes, health benefits and minimally processed food items, without additives and improvers, thereby satisfying the new consumer’s preferences and emerging life-trends related to ‘clean labelling’ of food products, healthy diets, and wellbeing.

In the present study, experimental breads were prepared under controlled conditions using plant ingredients identified in the prehistoric record and resembling some of the prehistoric and/or ancient breadmaking practices (ingredients, grinding tools, leavening agents) in an effort to establish in ongoing studies [32] a relevant foundation for the recognition of past culinary practices, as reflected in the archaeobotanical charred remains of breads produced from cereals, legumes, nuts and oilseeds. The current work aimed to evaluate the physicochemical characteristics of flours, doughs and breads made from these plant materials (grains, seeds, nuts), following their reduction into smaller particles (meal/flour) by prehistoric replica grinding tools and employing food preparation practices that were likely to be employed in prehistoric and/or ancient times. Besides simulating some aspects of prehistoric and/or ancient cuisine, the ultimate goal of our research is to use this information in the design and development of future bakery products with improved nutritional attributes and increased consumer acceptability.

## 2. Materials and Methods

### 2.1. Flours

**Figure 1:**
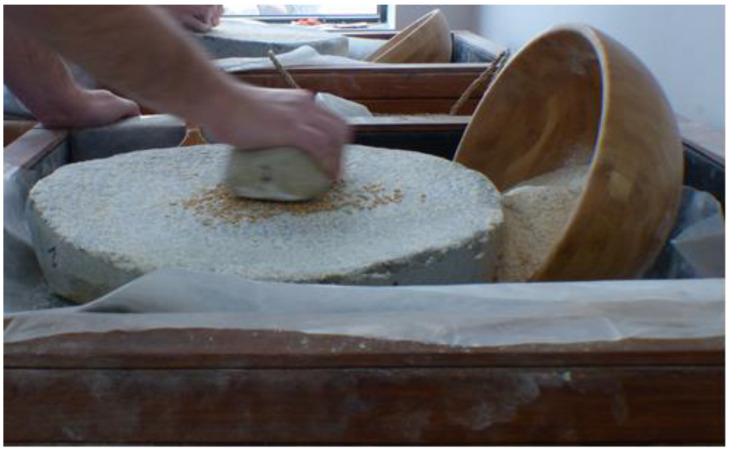
Grinding cereals for experimental foods in the context of the European Research Council (ERC) PlantCult project.

Five different plant raw materials—einkorn wheat (*T. monococcum*), barley (*Hordeum vulgare*), acorn (*Quercus* sp.), lentil (*Lens culinaris*), poppy seed (*Papaver somniferum*) and linseed (*Linum usitatissimum*)—were ground using prehistoric grinding tool replicas constructed for the PLANTCULT project (Figure 1), as described by Bofill et al. [33]. Specifically, einkorn grains were ground by three different types of grinding tools made from three different grinding stone materials (andesite, sandstone and granite) with three different grinding ways: (a) a grinding slab with a handstone of the “overhanging” type used in a back-and-forth reciprocal motion; (b) a grinding slab with a small handstone used in a back-and-forth reciprocal motion; (c) a grinding slab with a small handstone used in a circular and free-motion action. The rest of the grains and seeds were ground by the tool made from sandstone with the second way of grinding. The einkorn flour used for breadmaking and the study of the flour, dough and bread physicochemical properties was a mixture of the 9 flour samples ground by the three different stones and grinding ways mixed in equal amounts; this mixture was used as a representative flour sample prepared by the prehistoric grinding tool replicas. Two commercial flours from common wheat (*T. aestivum*), type T70 (white flour), and spelt wheat (*T. spelta*), whole flour, were also employed for breadmaking, whereas a commercial barley whole flour was also used for breadmaking and sourdough preparation. All the above three commercial flours were organic, provided by a local supplier (Doumos, Irinis Garden, Aridaia, Pella, Greece).

#### 2.1.1. Particle Size Distribution

Flour particle size distribution was determined by sieve analysis using 100 g of a weighed sample, which was passed through a series of sieves with pore sizes from top to bottom: 4-mesh (0.96 mm), 5-mesh (0.80 mm), 6-mesh (0.65 mm), 8-mesh (0.50 mm), 10-mesh (0.40 mm), 16-mesh (0.24 mm), 20-mesh (0.20 mm), 30-mesh (0.13 mm), 40-mesh (0.10 mm) and 70-mesh (0.063 mm). For particle size analysis of einkorn flour, all of the above sieves were used, whereas for the commercial flours of barley and common wheat, only the last six sieves were employed. After shaking for 15 min by a mechanical vibratory shifter, the amount of flour retained on each sieve (WS) was weighed, and the contents of retained fractions were calculated as the percentage of the initial (total, WT) flour weight as follows:

Retained fraction (%) = (WS/WT) × 100(1)

The cumulative percent passing through the sieves was calculated by subtracting the cumulative percent of retained fractions from 100%.

Cumulative percent Passing % = 100% − Cumulative Retained %(2)

The particle size parameters of d_50_ (median diameter) and d_90_ that are commonly used in the classification of ground materials were estimated, representing 50% and 90%, respectively, of the particles with diameters smaller than the specified values.

#### 2.1.2. Calorimetric Study of Starch Gelatinization Properties

The starch gelatinization properties of flours and flour mixtures (Table 1) used for breadmaking were studied by Differential Scanning Calorimetry (DSC) using a PL DSC-Gold calorimeter (Polymer Labs. Ltd., Epsom, UK). All flours obtained from the prehistoric grinding tools were passed through a coarse sieve (~1mm) to remove the coarse particles of husk and bran and thus increase the content of starch in the samples. Aqueous slurries of the sieved samples (about 25–30mg) containing 35% *w*/*w* solids were sealed hermetically into DSC aluminum pans. The pans were heated from 8 to 120 °C at a heating rate of 5 °C/min; samples were heated under a continuous flow of dry N_2_ gas (20 mL/min) to avoid moisture condensation during measurement. Three specimens from each flour preparation were analyzed by DSC. Parameters estimated from the DSC thermographs were the onset (T_o_) and peak (T_m_) starch gelatinization temperature, as well as the apparent starch gelatinization enthalpy (ΔΗ) calculated from the area of the endothermic (melting) peak, following calibration of the calorimeter with indium.

### 2.2. Sourdough

#### 2.2.1. Sourdough Preparation

**Figure 2:**
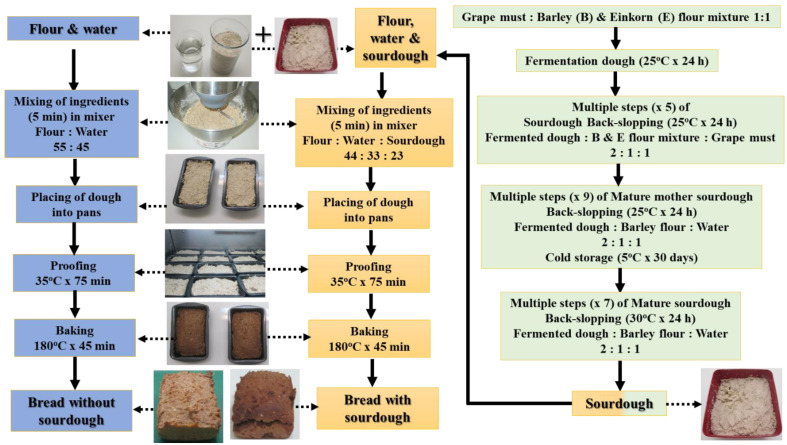
Flow chart of the protocol followed for mature sourdough production and breadmaking without and with sourdough usage; the flour (or flour mixture) formulation used for breadmaking without and with sourdough is given in Table 1.

The sourdough used for breadmaking was prepared for project PLANTCULT by the traditional method of spontaneous back-slopped sourdough (Type I); all the preparation steps are presented in detail in Figure 2. For the first fermentation step, a mixture of barley and einkorn wheat flour (barley:einkorn 1:1) with grape must (grape must:flour mixture 1:1 *w*/*w*) was used, following a recipe provided in a modern sourdough preparation book for the wider public [34]. The use of leavened bread is mentioned in the Hippocratic corpus, while the use of a diluted form of grape juice or of the by-product of alcoholic fermentation of grapes for breadmaking are mentioned in ‘Geoponika’, dated to the 10th c. A.D. [35]. Following fermentation at room temperature for 24 h, the mother sourdough was propagated five times by back-slopping steps using the barley and einkorn flour blend mixed with grape must, resulting in a mature mother sourdough. These fermentation steps were followed by further multiple (9) back-slopping steps at room temperature using barley flour, water and an amount of the mature sourdough; this mature sourdough was stored at 5 °C until it was used for breadmaking. Seven back-slopping steps using barley flour for the sourdough ‘refreshment’ were performed every 24 h to propagate the mature sourdough before breadmaking (Figure 2); fermentation during refreshments was carried out in closed vessels under controlled laboratory conditions in an incubator (Sanyo Incubator, MIR-154, SanyoElectric Co. Ltd., Ora-Gun, Gunma, Japan) at 30 °C for 24 h. The dough yield (DY) was defined as:

DY = (Flour weight + Water weight) × 100/Flour Weight(3)

and it was 200 for these back-slopping steps during the making of barley-based flour sourdough.

#### 2.2.2. Microbiological and Physicochemical Characteristics of Sourdough

For the determination of colony forming units (CFU/g) in mature mother sourdough and the final back-slopped barley-based sourdough used for breadmaking, 10 g portions of sourdough were homogenized with 90 mL NaCl (0.99%, *w*/*v*), followed by decimal dilutions in the same saline solution. Enumeration of microorganisms was carried out using the pour plate technique. Lactic acid bacteria were enumerated on De Man Rogosa and Sharpe agar (MRS agar) (Merck KGaA, Darmstadt, Germany) containing natamycin, following incubation at 30 °C for 3 days, while for enumeration of yeasts, Yeast Malt Agar (YM agar) (Sigma-Aldrich, St. Louis, MO, USA), containing tartaric acid was used after incubation at 25 °C for 5 days.

For pH and total titratable acidity (TTA) determinations, 10 g of sourdough were mixed with 90 mL of sterile distilled water, and the resultant suspension was kept under continuous stirring during both measurements. The pH was measured with a Bante 210 pH meter (Bante Instruments Co., Shanghai, China), and the TTA was determined by titration of the sourdough suspension with 0.1 N NaOH until a final pH of 8.4; the TTA values were expressed as mL of 0.1 N NaOH/g of sourdough.

### 2.3. Doughs

#### 2.3.1. Dough Preparation

For the study of dough rheological properties, flours obtained with the prehistoric grinding tools were passed through a coarse sieve (~1 mm) to remove all coarse particles of husk and bran and thus increase their homogeneity and improve their water retention capacity. Dough samples from all tested flours used for breadmaking (Table 1) without sourdough were prepared by mixing the flours with tap water (flour:water 50:50 *w*/*w*), and followed by hand-kneading for 5 min. Before rheological testing, all dough samples were wrapped with a plastic membrane to avoid water loss and rested at room temperature for 20 min, allowing uniform moisture distribution and dough matrix relaxation before testing. The dough preparation procedure for each formulation was repeated in triplicate.

#### 2.3.2. Dough Rheology

Oscillatory measurements of doughs were performed by a rotational Physica MCR 300 rheometer (Physica Mess-technic GmbH, Stuttgart, Germany) using a parallel plate geometry (50 mm diameter and 2 mm gap) with a solvent trap to avoid moisture loss during measurements; the plate had a sanded surface to prevent slippage of the measuring fixture. The temperature was regulated at 20 °C by a controlled temperature peltier system (TEZ 150P/MCR) with an accuracy of ±0.1 °C. After loading, the dough sample was left to further rest in the geometry for 15 min prior to measurement. Oscillatory measurements were performed, at which the storage (or elastic) modulus, G′; loss modulus, G″; and complex viscosity, η*, at a strain level of 0.01%, were monitored over an angular frequencies range of 0.1–50 Hz. The data of the rheological measurements were analyzed with the supporting rheometer software US200 V2.21.

### 2.4. Breads

#### 2.4.1. Bread Formulations

Breads were made from einkorn, spelt, barley and common wheat flours alone as well as from flour mixtures of einkorn with another grain or seed flour, i.e., spelt, acorn, lentil, barley, poppy seed and linseed (einkorn: other flour in an 80:20 ratio), with the addition of sourdough; all bread formulations are shown in Table 1. Einkorn was employed as the primary flour (larger proportion in the mixture), since it was a very important species used in prehistoric Greece [36,37,38]; moreover, it has previously been found that it can have a relatively acceptable breadmaking performance, especially when sourdough is included to an einkorn bread formulation [39,40,41]. Additionally, a control bread formulation made from einkorn flour alone without sourdough was prepared for comparison. The back-slopped barley-based sourdough (originating from the mature mother sourdough) was used for breadmaking (Figure 2) at the level of 20% *w*/*w* on a flour basis (DY 200); i.e., the amount of flour from sourdough was 20g in 100g of total flour in the bread formulation. The level of added water in all dough formulations, including the sourdough water amount, was 80% (flour basis); the same water level was also added to the control sample.

#### 2.4.2. Breadmaking Process

For the breadmaking process, flours obtained with the prehistoric grinding tools were used without removal of the coarse particles of husk and bran in order to closely resemble the prehistoric practices. Actually, pieces of cereal bran and husk embedded in a cereal-based amorphous matrix (gelatinized starch) have been identified in several archaeological sites in Southeastern Europe, indicating that breadmaking and/or porridge making from cereal grains were possibly widespread food preparation methods in prehistoric times, by employing relatively simple processing tools and practices [9].

The flow chart presented in Figure 2 describes the major steps of breadmaking followed in the current study. Breads were prepared by mixing sourdough, flour and water for 15 min with a mixer (KMM023, Kenwood Major Titanium, Kenwood Ltd., Havant, UK) at medium speed and room temperature. The composite dough (150 g) was placed in pans (three pans for each dough formulation) and proofed under controlled temperature and relative humidity (RH) conditions in an incubator (Sanyo Incubator, MIR-154, SanyoElectric Co. Ltd., Ora-Gun, Gunma, Japan) at 35 °C and 100% relative humidity (RH) for 75 min. Baking was performed in an oven (air-o-stream combi oven, Electrolux Professional SpA, Pordenone, Italy) under controlled temperature (180 °C) and RH (100%) conditions for 45 min; the exhaust valve of the oven was opened in the last 10 min of baking to remove excess humidity from the chamber. The breadmaking procedure for each bread formulation was repeated in triplicate. Analyses of quality parameters of the breads were performed after cooling them down to room temperature for 1.5 h, and the reported values of tested quality attributes are mean values from the three breadmaking processes.

#### 2.4.3. Bread Quality Characteristics

Large deformation mechanical properties of the experimental breads were examined by a puncture test using a Texture Analyser (TA-XT2i, Stable Micro systems, Godalming, Surrey, UK) calibrated with a 5 kg load cell. The bread loaves were compressed with a spherical probe (0.635 cm diameter) up to rupture of the crust at a crosshead speed of 0.4 mm/s. Hardness of the crust was taken as the peak force of the force-displacement curve; two values obtained from the same loaf were averaged, and two loaf breads from each breadmaking process were averaged into one replicate.

The volume of the bread loaves was determined with a homemade volume meter made from plexiglass and based on the rapeseed displacement method [42]. Bread loaves were weighted, and the specific volume was calculated as the ratio of volume/bread weight. For each bread formulation, the measurement was carried on three loaves (one from each breadmaking process).

### 2.5. Microscopy and Changes in Starch Physical State during Breadmaking and Bread Storage

Suspensions in ethanol of einkorn and commercial barley flours, barley sourdough, einkorn dough without and with sourdough, as well as crumb of fresh einkorn bread without and with sourdough were prepared under stirring. Aliquots of suspensions were taken by aspiration and examined using an Olympus BX51 microscope (Japan) equipped with dry lenses, a microscope digital camera Olympus DP70 and the Olympus micro DP70 software. The microscopic observation was carried out after staining of starch with iodine solution in bright field and cross polarized light in order to observe the starch granules and the changes in starch microstructure during dough preparation and breadmaking in the presence or absence of sourdough. At least ten captures for each sample were taken.

Changes in starch physical state upon breadmaking and bread storage were also examined by DSC analysis of the crumb of fresh and staled einkorn bread without and with sourdough; for the staling events in the starch matrix, bread loaves were stored in sealed polypropylene bags at 3 °C for 6 days. Before analysis, crumb samples from fresh and stored bread were lyophilized and then ground into fine powder using liquid nitrogen. Aqueous slurries of the lyophilized samples (about 25–30 mg) containing 35% *w*/*w* solids were hermetically sealed into DSC aluminum pans and heated from 8 to 120 °C at a heating rate of 5 °C·min^−1^. Three crumb specimens of each bread preparation from all the breadmaking processes were tested by DSC. The onset melting temperature (T_o_^RET^), the peak (T_m_^RET^) melting temperature and the apparent melting enthalpy (ΔH^RET^) of the retrograded starch were determined.

### 2.6. Statistical Analysis

All physicochemical parameters of flours were tested in triplicates. Mean values of dough parameters, loaf specific volume and crumb retrogradation parameters were the average from three dough specimens, bread loaves and crumb samples, respectively (one from each dough or bread making procedures). For crust hardness, firstly, values from two different points of the same loaf were averaged, and the mean values presented in this study were obtained from two different breads that averaged into one replicate of the three breadmaking repetitions.

Statistical analyses were performed by the IBM SPSS statistical software (version 23.0, IBM Corp., Armonk, NY, USA). All parameters of flour, dough and bread properties were analyzed by a one-way ANOVA, according to a generalized linear model, examining the effect of flours and their mixtures at all breadmaking stages. Differences between mean values were compared using the Tukey’s test at a = 0.05 significance level.

## 3. Results and Discussion

### 3.1. Flour Properties

**Figure 3:**
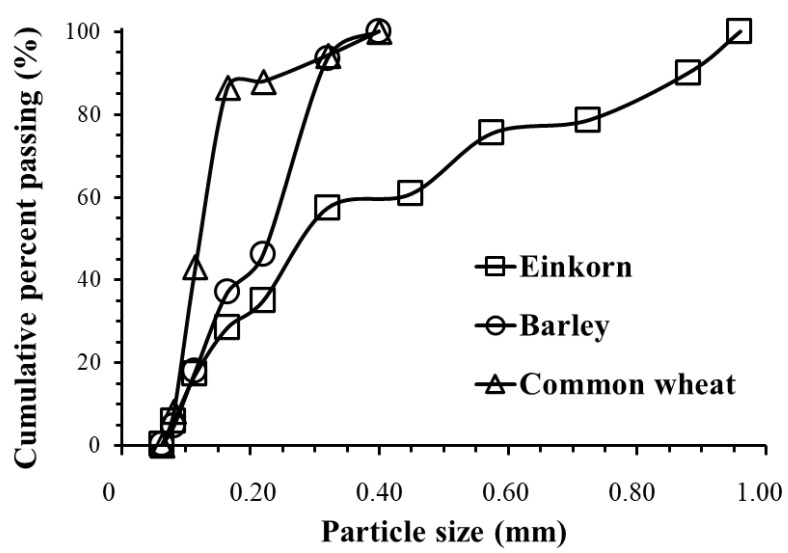
Cumulative distribution of particle size of einkorn, barley and common wheat flours.

Analysis of sieving classifies the flour particles by size as well by shape. The particles of flour are usually spherical-like, such that their diameters correspond to the sides of the square sieve opening. Particle size cumulative distribution curves of einkorn, barley and common wheat flour are presented in Figure 3. Sieve analysis of flour particle size distribution showed that 40% of the particles of einkorn flour had a size >400 μm, while the sizes of all particles of commercial barley and common wheat flour were smaller than this size. Specifically, the d_50_ and d_90_ values of einkorn, barley and common wheat flour were 287, 228, 123 μm and 879, 313, 252 μm, respectively. Thus, einkorn flour exhibited the largest particle size at all particle size distribution ranges among the three tested flours since einkorn grains were ground by prehistoric grinding tool replicas (stone grinding), in contrast with the other two commercial flours, which were finely ground by industrial mills. Additionally, between the two commercial flours, barley flour had larger particles as it was a whole flour compared with those from common wheat, which was a white flour (70% extraction rate); it is well-known that whole flours include bran and thus have higher mean particle size compared with flours originating mostly from the endosperm of cereal grains (white flours).

According to previous studies, the particle size of flours significantly affects the rate of water absorption during dough making, as fine particles absorb water faster due to their greater surface area [43]. Smaller flour particles from some starchy grains such as quinoa were also found to have an impact on starch gelatinization properties [44], i.e., the finer flours exhibited lower starch gelatinization temperatures. Moreover, it has been shown that flour particle size largely affects cereal flour dough rheological behavior, with doughs from coarser barley flour (d_50_ = 350 μm) exhibiting increased stiffness and resistance to deformation and flow compared with that of a fine (d_50_ = 200 μm) barley flour preparation [27]. However, in our study, the effect of particle size on dough rheological and thermal properties cannot be clearly unraveled, since the different tested flours originated from different grain species, and their compositional differences can certainly have a stronger impact on flour and dough functional properties. It is worth noting that flour particle size can have a significant effect on the starch digestibility of bakery products and thus can have an impact on postprandial glycemic responses. Our previous studies using an in vitro assay simulating the human digestion process have shown a lower starch degradation by digestive enzymes for rusks made from a coarse barley flour compared with the products made from a fine flour (37% vs. 53% after 5 h of digestion) [27]; therefore, coarse flours such as those ground by stone mills, as employed in the present study, can lead to a better attenuation of glucose blood levels compared with products made by commonly used wheat fine flours. Moreover, whole ancient grain flours are rich sources of dietary fiber and other bioactive compounds, such as antioxidants [21,22,23].

**Figure 4:**
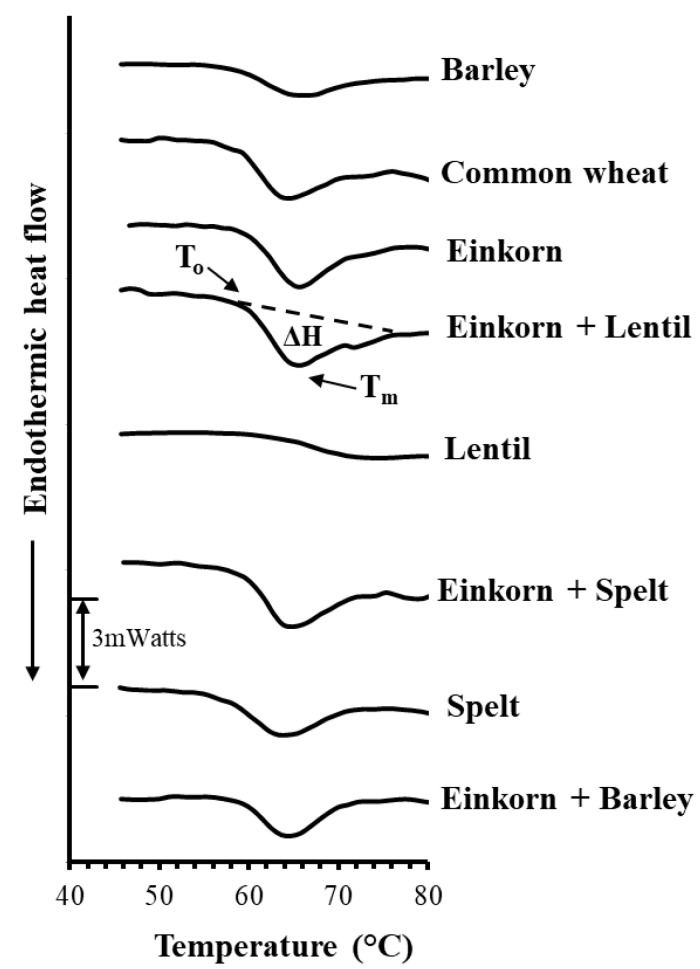
Representative Differential Scanning Calorimetry (DSC) thermographs of slurries (35% *w*/*w*) of the flour samples used for breadmaking (heating rate 5 °C/min): T_o_, onset starch gelatinization temperature; T_m_, peak starch gelatinization temperature; ΔH, apparent starch gelatinization enthalpy; notation of samples as in Table 1.

The DSC thermograms of the slurries (35% *w*/*w* solids) of tested flour samples showed the typical endothermic peak of starch gelatinization at around 56.0–80.0 °C (Figure 4), which is the usual temperature range at which this phase transition occurs, at similar water levels to those used in the present study [45]. The endothermic peak is attributed to absorbed thermal energy, resulting in the breaking of the hydrogen bonds between adjoining starch polymeric chains existing in double helical conformations. The swelling of starch granules in heated aqueous dispersions usually starts at a temperature corresponding to the onset temperature (T_o_) of this endothermic transition and the disruption (melting) of starch molecular orders (mostly double helical structures of amylopectin) upon gelatinization occurs at the peak temperature (T_m_) (Figure 4); the area under the endothermic peak expresses the apparent melting enthalpy (ΔΗ), reflecting the net amount of heat, required for the disruption of short- and long-range molecular orders in the starch granules of the heated sample.

Lentil flour dispersions exhibited the highest T_o_ (64.7 °C) and T_m_ (74.0 °C) and the lowest ΔH (0.9 mJ/mg) values among all tested flour samples, implying higher resistance of this legume starch towards gelatinization and a relatively lower amount of double helical structures (primarily involving the amylopectin component) compared with those of cereal starches (Table 2 and Figure 4). It is well known that the thermal transition temperatures of cereal and legume flours differ among different species and are influenced by water, protein and amylose content, level and type of helical structures in the starch granules, distribution of amylopectin branch chains, and the presence of monoacyl lipids, which can complex with amylose chains into single helical structures during starch gelatinization [45,46]. Αmylopectin plays a major role in starch granule crystallinity; however, in the case of high amylose content starch, the melting temperature of crystalline regions increases, the endotherm broadens and there is also a change in the apparent gelatinization enthalpy of the starch component [47]. Therefore, the differences in T_o_, T_m_ and ΔH values among lentil seed and cereal grain flours observed in the current study could be attributed to the higher amylose content of starch, ~30–40%, and the lower total starch concentration of flour, ~50%, for legumes than typical cereal grains (einkorn, spelt and barley), which have ~20–25% and 60–70%, respectively [23,46,48,49,50,51,52]. Among cereal grains, spelt showed the lowest T_o_ temperature (56.2 °C) followed by the common wheat (57.6 °C), while T_o_ values for flours of barley, einkorn and its mixture with lentil, spelt and barley displayed higher values, ranging from 58.9 to 59.4 °C (Table 2). For cereal flours and flour mixtures with einkorn as their major component, the T_m_ values were similar, varying in the narrow range of 61.6–65.7 °C, while their gelatinization enthalpy ranged between 3.2 to 5.6 mJ/mg of flour (Table 2). It seems that the gelatinization properties of flour mixtures were not largely influenced by enrichment with the secondary flour, being similar to those of the base flour (einkorn), since fortification with the secondary flour in the flour mixtures was in a small proportion (20% *w*/*w*). Moreover, barley flour showed significantly lower apparent enthalpy and higher onset temperature of gelatinization compared with common wheat flour (Table 2), probably due to the presence of a higher amount of non-starch polysaccharides (cell wall materials from bran and endosperm) in the whole barley flour. In accordance with our findings, Tester and Sommerville [53] demonstrated that the presence of non-starch polysaccharides limited water availability and reduced the leaching of amylose from starch and hence the swelling factor of starch granules during gelatinization, resulting in an increase in the apparent T_o_ and a reduction in ΔΗ.

### 3.2. Microbiological and Physicochemical Characteristics of Sourdough

The predominant microflora in sourdoughs are lactic acid bacteria (LAB), while the number of yeasts is limited [54]. In our study, the number of cells of lactic acid bacteria were much higher (9.8 log CFU/g) than that of yeasts (6 log CFU/g) in the sourdough added to bread dough. The numerous refreshment steps of the original sourdough aimed to establish a final sourdough preparation for breadmaking with increased LAB cell density and a rather suppressed yeast population. Indeed, it appeared that the conditions of the multiple refreshment steps did not allow propagation of yeasts and mostly resulted in the domination of LAB. The pH of the final sourdough dropped to 3.8, whereas the TTA reached a value of 11.7 mL NaOH 0.1 N/10 g of sourdough; these values can be considered as indices of a well-developed sourdough system [55]. Other researchers reported similar LAB and yeast cell densities, pH values, and TTA levels for spontaneous back-slopped barley sourdoughs, as well as for spontaneously fermented durum-wheat-based sourdough when must grape was employed as an added ingredient to properly ‘drive’ the fermentation process by providing fermentable sugars and competitive microflora, as well as to prevent undesirable microbial deviations [56,57].

### 3.3. Dough Rheological Properties

**Figure 5:**
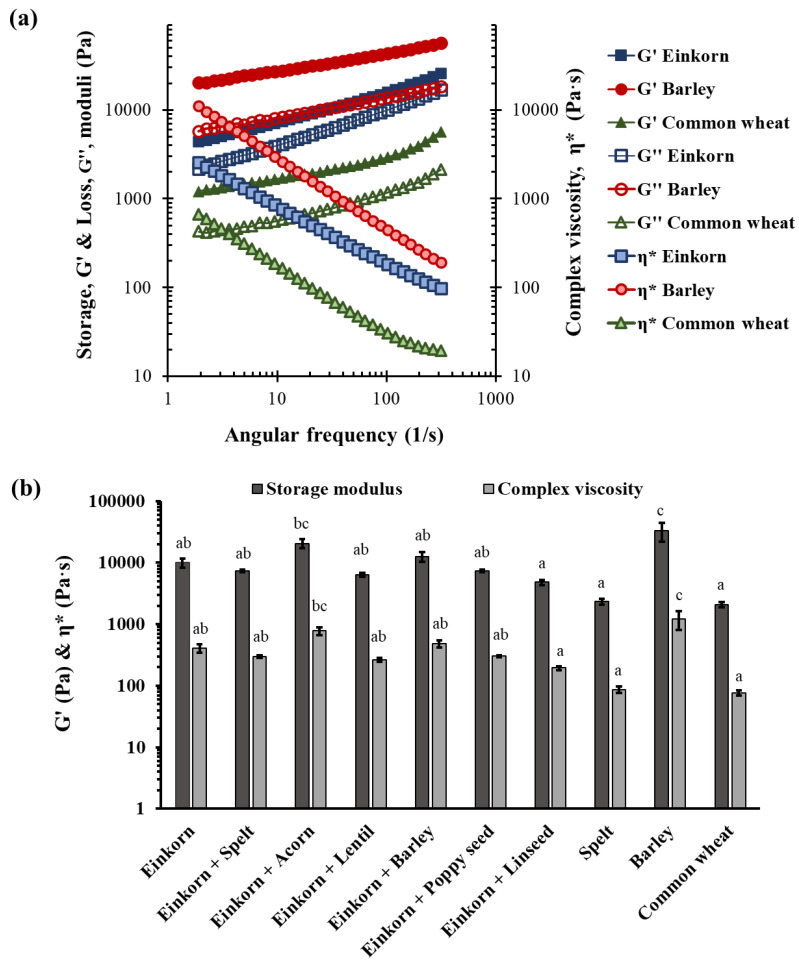
Representative mechanical spectra of doughs (flour:water 50:50 *w*/*w*) of the flour samples used for breadmaking (**a**) and the derived storage modulus, G′, and complex viscosity, η*, at 30 1/s angular frequency (**b**); strain 0.01%, 20 °C. Values followed by the same letter for the each specified rheological parameter are not significantly different (*p* > 0.05, Tukey’s test); notation of samples as in Table 1.

The rheological properties of flour doughs are influenced by many factors, such as dough ingredients (composition), temperature, water uptake and type of mixing, but the most important is the type of flour used [58]. Figure 5a illustrates three representative mechanical spectra of einkorn, barley and common wheat doughs. All dough formulations showed the typical solid, elastic-like behavior of wheat and non-wheat-based doughs, with the elastic modulus being greater than the loss modulus over the whole frequency range and both moduli being slightly dependent on frequency [59,60]. The G’ and η* values that were obtained from the frequency sweep test ranged between 2080–33400 Pa and 76.8–1214.0 Pa·s, respectively (Figure 5b). The barley dough exhibited significantly (*p* < 0.05) higher elastic modulus and complex viscosity values compared with all doughs made from any wheat species flour (einkorn, spelt and common wheat), suggesting that the barley dough was the most resistant to deformation and flow. Barley non-starch polysaccharides, such as β-glucans and arabinoxylans, can provide a composite dough with increased structural strength, stiffness and viscosity because of their ability to bind large amounts of water [61,62,63]. On the other hand, the different wheat species, both ‘modern’ (common wheat) and ‘ancient grains’ (einkorn and spelt wheat), produced doughs with similar rheological parameters (*p* > 0.05).

Τhe supplementation of einkorn wheat dough with spelt, barley, acorn, lentil, poppyseed and linseed flour did not result in any significant (*p* > 0.05) change in its rheological behavior (Figure 5b). Nevertheless, the incorporation of linseed into einkorn wheat dough led to a small decrease of dough elasticity, probably due to its lubricating action, while inclusion of acorn flour resulted in a slight strengthening of the dough; thus, the most elastic and viscous einkorn doughs were those supplemented by acorn flour, as indicated by the respective values of their rheological parameters. Our findings are in accordance with those of Beltrão Martins et al. [64] and Korus et al. [65] who studied the influence of acorn flour on rheological properties of gluten-free dough; these researchers found that the incorporation of acorn flour into gluten-free dough formulations resulted in an increase of G’ values.

It is worth noting that high dough elasticity and viscosity implies high resistance of dough to deformation (high G’ value) and flow (high η* value) and cannot necessarily be related to improved dough and bread textural properties [66]. High viscosity and elasticity of a wheat flour dough could also be linked with limited dough expansion and insufficient retention of the incorporated gas cells during mixing and/or from sourdough fermentation, thereby resulting in a more compact crumb macrostructure and lower bread volume.

### 3.4. Evaluation of Bread Quality Characteristics

Quality characteristics of breads, such as appearance, loaf volume and bread texture, are major determinants of the product acceptability by consumers. Despite the expected positive health implications of ‘ancient’ cereal grains consumption, their involvement in bread production usually leads to doughs characterized by high tenacity and low extensibility, while the resultant breads exhibit reduced loaf volume [67,68]. However, a small number of spelt, emmer and einkorn cultivars were found to have favorable gluten characteristics for good baking performance [12]. In the current study, the breads made were based on ‘ancient’ grains enriched with other ‘ancient’ plant derived ingredients—which were often used in food products, including breads, in the prehistoric and ancient past by humans [3,4,6,7,8,9,15,16]—by employing stone grinding tools [13,33] similar to those used in prehistoric times and sourdough as the only leavening agent; leavened bread was likely used in ancient cuisine as well [35].

**Figure 6:**
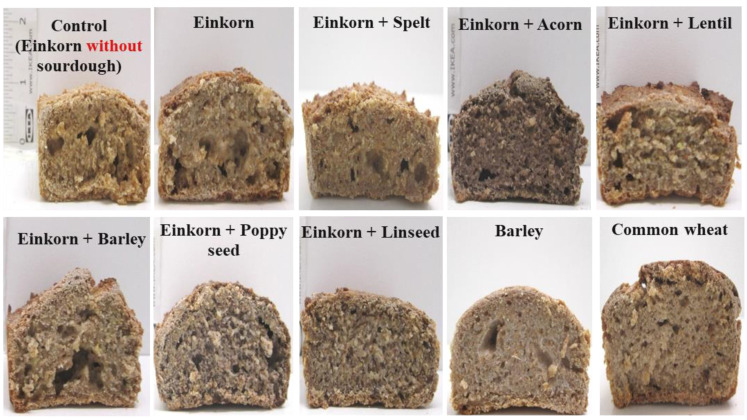
Appearance of a cross-section of breads; all breads made with sourdough except control; notation of samples as in Table 1.

The appearance of the loaf cross-sections of all bread formulations is given in Figure 6. Macroscopically, it was shown that the crumb macrostructure of ancient grain-based breads was inferior compared with that of the commercial common wheat flour. Thus, breads from einkorn and its mixtures with other plant materials had uneven gas cell distributions exhibiting large pores. The latter implies gas cell coalescence during breadmaking, probably due to the weak protein gel network formed upon dough mixing and kneading, as well as the presence of much larger amounts of bran particles in the formulation compared with common wheat bread; bran particles can also weaken the gluten network by dilution, competition for water absorption and interruption of its continuity. Additionally, bread from commercial barley flour showed a relatively more cohesive and compact crumb macrostructure than the other formulations, which is possibly attributed to the presence of non-starch polysaccharides (arabinoxylans and β-glucans) that can largely increase the viscosity and elasticity of the dough, resulting in prevention of extensive dough raising; this observation is in agreement with the findings from the mechanical spectra of dough formulations, in which barley dough showed the highest G΄ and η* values (Figure 5) among all the tested preparations. Moreover, bread enriched with acorn flour exhibited the darkest crumb and crust color among the samples due to the presence of this dark-coloured ingredient (Figure 6), indicative of the presence of a high concentration of phenolic compounds in the raw material.

**Figure 7:**
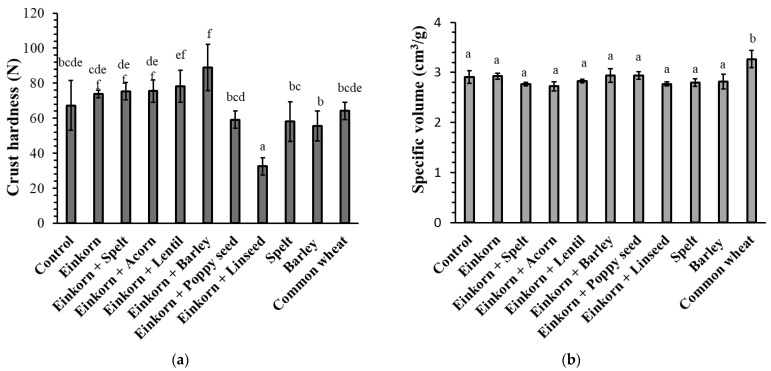
Crust hardness (**a**) and specific volume (**b**) of breads; control formulation is einkorn bread without sourdough. Values followed by the same letter are not significantly different (*p* > 0.05, Tukey’s test); notation of samples as in Table 1.

Sourdough inclusion in einkorn bread formulation did not seem to affect the crust hardness (Figure 7a); thus, the crust hardness of einkorn bread with sourdough did not significantly differ (*p* > 0.05) from that of the control bread (einkorn without sourdough). On the other hand, incorporation of linseed into the einkorn bread decreased (*p* < 0.05) the crust hardness significantly; thus, einkorn bread fortified with linseed had the lowest crust hardness among all tested samples. In agreement with our findings, Marpalle et al. [69] have also observed that bread’s softness increased with increasing level of flaxseed flour added in fortified breads. Moreover, poppyseed inclusion in einkorn bread formulation resulted in a softer crust than the other einkorn-based breads, although this effect was not significant (*p* > 0.05) (Figure 7a). Soft crust texture of bread formulations fortified with these oilseeds could be attributed to the high fat content of linseed [70,71,72,73] and poppyseeds [74,75] (~40–45% fat), with the lipids acting as lubricants, decreasing the crust hardness of the final product. Instead, the inclusion of stone-ground barley in einkorn bread formulation resulted in the highest crust hardness value among all samples (Figure 7a).

Loaf volume is commonly considered as the most important indicator of bread quality. The bread from common wheat flour had significantly higher loaf specific volume compared with all other bread samples (Figure 7b). The higher volume is ascribed to the unique viscoelastic properties of gluten in common wheat flour resulting in the development of a strong protein cross-linked network in dough, which leads to retention of gas cells during proofing and baking. The presence of high amounts of bran particles or non-starch polysaccharides in einkorn, spelt and barley-based breads, on the other hand, can lead to a loaf volume reduction, since these carbohydrate polymeric materials can compete with proteins and starch for water absorption-retention and thus interrupt the continuity of a well-developed protein gel network during dough mixing, as well as the gelatinized starch in the dough continuous phase throughout baking; the presence of all these non-starch polysaccharides (soluble as well as insoluble particles) also weakens the continuity of the composite gluten-starch network formed upon baking of the dough. Similarly, Geisslitz et al. [12], comparing breads made by ‘ancient’ wheat species (einkorn, spelt and emmer) to those of common wheat and durum wheat, reported that among the five wheat species, the common wheat flour gave the highest loaf volume. According to these researchers, a high molecular weight glutenin subfraction, namely glutenin macropolymer (GMP), is positively correlated with dough water absorption and bread volume, pointing to a strong impact of protein quality (glutenin fraction) on breadmaking performance; among the different species of wheat, the GMP contents of common wheat, spelt and einkorn were ~0.8, 0.6 and 0.3 g/100g of whole meal flour, respectively.

### 3.5. Starch Physical State of Fresh and Stored Bread

**Figure 8:**
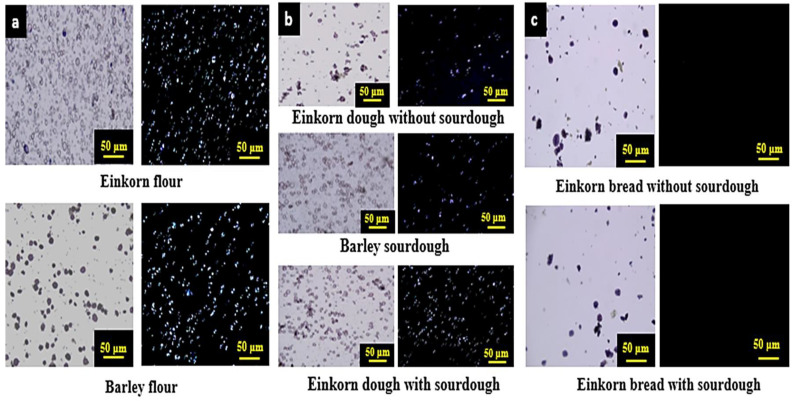
Optical micrographs of starch granules from einkorn and barley flours (**a**), barley sourdough and einkorn dough with and without sourdough (**b**), and crumb of einkorn fresh bread with and without sourdough (**c**), stained by iodine solution and observed under bright-field light (left pictures) and cross-polarised light (right pictures).

Iodine staining of the starch granules of einkorn and barley flour revealed the typical bimodal size distribution and the characteristic oval and round shape of wheat and barley starch granules (Figure 8a-left). Additionally, optical birefringence of both flours was evidenced in the cross-polarized micrographs, reflecting the ordered structures in the starch granules at a molecular level (Figure 8a, right).

As expected, shape, integrity, size and birefringence of einkorn and barley starch granules were preserved in the dough and sourdough preparations (Figure 8b). It is worth noting that some starch granules in the einkorn control dough (without sourdough) seemed to be clustered (Figure 8b, top). Other researchers studying the dough microstructure have found, by staining both protein and starch, that these components are not evenly distributed in the dough, and there are some regions of the dough where several starch granules are gathered [76]; it seems that the gluten network fills the space between the water-fused starch granules. In contrast, starch granules are more evenly distributed in barley sourdough (Figure 8b, middle); most likely, the network of the barley storage protein is weaker and does not hinder a homogeneous distribution of starch granules into the water phase. A more homogeneous distribution of starch granules was also favored in the einkorn dough specimens when barley sourdough was added (Figure 8b, bottom); possibly, sourdough dilutes and interrupts the continuous native gluten gel network in the einkorn dough system.

During baking, the rise of temperature up to about 95 °C in the crumb and the level of added water to the dough allows the gelatinization of starch to occur. Therefore, starch granules appeared larger than those in flour and dough due to extensive swelling and loss of their oval or round shape; nevertheless, the swollen granules still retained their identity (Figure 8c, left). The loss of granular integrity is probably attributed to the melting of starch crystallites and leaching of starch molecules (mostly amylose) from the swollen granules, which largely occur upon starch gelatinization. Moreover, the presence of sourdough in einkorn bread did not seem to affect the starch microstructure in the crumb. As expected, bread crumb exhibited almost no birefringence, since starch granule swelling upon gelatinization was accompanied by loss of the ordered structure of starch molecules, i.e., the melting of the crystalline domains initially present in native starch granules (Figure 8c, right).

Heat-moisture mediated disruption of the ordered structures in granular starch (gelatinization) is generally a prerequisite for its utilization because it changes the rheological properties of the system and has a major influence on the functionality and digestibility of starch-containing products [45], e.g., gelatinized starch largely contributes to the formation of a fine porous crumb structure in bread. However, formation of new structures (intra- and intermolecular associations) upon cooling and storage of starch systems, named as retrogradation, may be detrimental to end-product quality (texture changes), i.e., starch retrogradation significantly contributes to the hardening of bread crumb upon the staling of bread and other bakery items [77]. Starch retrogradation involves reassociation of the polymeric chains, creation of a new molecular order (mostly double helices of the amylopectin outer chains) among starch chains, and crystallization of double helical aggregates. The most common method to monitor these phenomena and to probe the development of the various structural domains in a starchy matrix is calorimetry.

**Figure 9:**
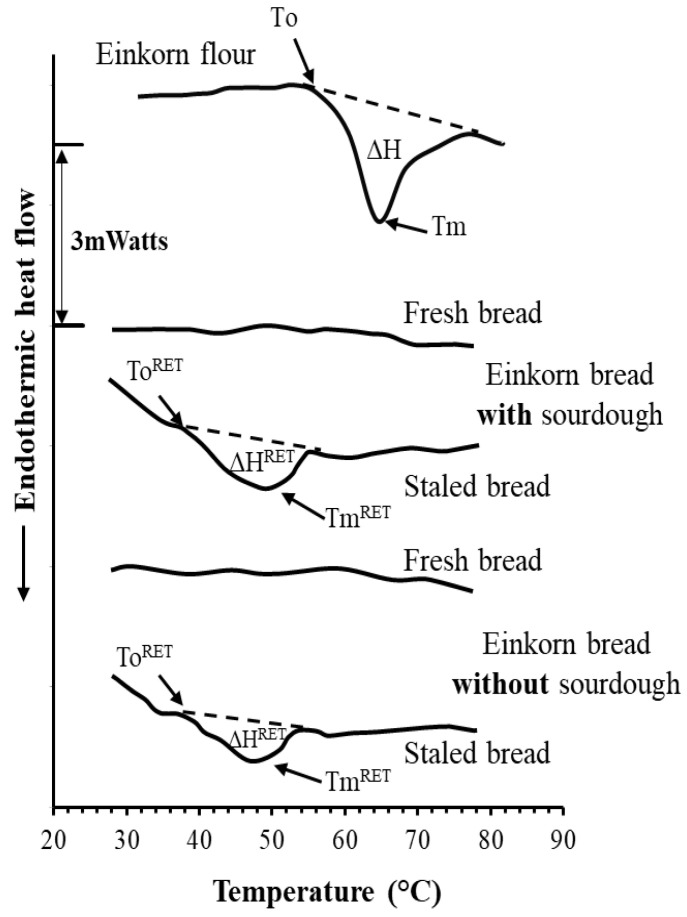
Representative DSC thermographs of slurries (35% *w*/*w*) of einkorn flour and crumb of fresh and staled (stored at 3 °C for 6 days) bread without and with sourdough (heating rate 5 °C/min). T_o_^RET^, onset of melting temperature of retrograded starch; T_m_^RET^, peak melting temperature of retrograded starch; and ΔH^RET^, melting enthalpy of retrograded starch.

As previously mentioned, the DSC thermograms of einkorn flour slurries (35% *w/w* solids) showed the typical endotherm peak of starch gelatinization at around 55–80 °C, with the peak starch gelatinization temperature (T_m_) occurring at about 65 °C, while the enthalpy (ΔH) required for the disruption (melting) of the ordered structures in native starch is estimated by the area under this peak (Figure 4 and Figure 9). This endothermic peak is not found in the DSC thermograms of fresh bread crumb (Figure 9) because of disordering (gelatinization) of starch molecules upon baking; this observation is consistent with the total loss of granule birefringence viewed by cross-polarized light microscopy of freshly prepared bread (Figure 8c, right). However, an endothermic peak (staling endotherm) at a lower temperature range (38–55 °C), with a peak temperature of around 50 °C, eventually appears on the DSC thermograms of bread crumb (35% *w*/*w* solids) after storage for long time (6 days) (Figure 9). The endothermic peak of staled bread is attributed to the melting of the retrograded amylopectin fraction (re-organized double helices of the outer short chains in the amylopectin molecules); this starch component retrogrades slowly upon storage of starchy aqueous systems, and the area under the endothermic peak continuously increases with storage time [45]. Specifically, this endothermic transition represents the melting of retrograded amylopectin, with ΔH^RET^, T_o_^RET^ and T_m_^RET^ corresponding to the apparent melting enthalpy and the onset and peak temperature, respectively, for melting of the re-ordered starch chains (Figure 9). The inclusion of sourdough in bread formulation did not significantly (*p* > 0.05) affect these three parameters; the respective values of ΔH^RET^, T_o_^RET^ and T_m_^RET^ were found to be 2.0 ± 0.6 mJ/mg (of dry bread), 38.0 ± 0.3 °C and 49.1 ± 0.6 °C for stored einkorn bread without sourdough, and 2.7 ± 0.7 mJ/mg (of dry bread) 38.1 ± 0.3 °C and 48.5 ± 0.9 °C for bread samples with sourdough.

In a previous study, the evolution of ΔΗ^RET^ in common wheat bread (crumb) upon storage (0–5 days) was examined, and it was found that the typical endothermic peak of melting of retrograded starch appeared at approximately 40–60 °C with T_m_^RET^ at 51 °C, and the ΔΗ^RET^ value for the 5th day of storage was 2.7 mJ/mg of dry bread [78]; these results are in agreement with the findings of the current study. Therefore, bread made from einkorn wheat seemed to follow a similar starch retrogradation behavior to that made from common wheat. This may imply that replacement of common wheat with einkorn in bread formulations may not have an adverse impact on the shelf-life of such a composite bakery product.

## 4. Conclusions

In the present study, the physicochemical properties of flours, doughs and breads made by using ancient plant ingredients (grain, nuts and seeds) as raw materials and adopting prehistoric grinding tool replicas for flour milling and sourdough making by multiple back-slopping steps as a bread leavening process were investigated. The research findings indicated that the particle size of einkorn flour ground with prehistoric-like stone mills was largely higher than it was for commercial fine flours of barley and common wheat. The starch gelatinization properties among cereal-based (spelt, barley, common wheat) flours were similar, while lentil flour had higher gelatinization temperature and lower enthalpy values. Overall, variations in the thermal, rheological and textural properties of the tested flours, doughs and bread formulations were observed among the different plant genera. More specifically, the barley flour made more elastic and viscous doughs compared with those containing any of the various wheat species used (einkorn, spelt and common wheat); additionally, inclusion of acorn into einkorn-based dough formulation resulted in similar rheological behavior with that of barley dough. Among the different wheat species tested, no significant variations in dough rheological properties were noticed. Similarly, the various tested cereal genera (barley and wheat species) did not differ in bread crust texture characteristics. However, fortification of einkorn bread with stone-grounded barley and linseed flour resulted in an increase and decrease in crust hardness, respectively. As expected, common wheat exhibited the highest loaf-specific volume among all bread formulations. On the other hand, the addition of barley-based sourdough into einkorn dough formulation did not affect either the textural properties and loaf volume, nor the extent of starch retrogradation of the final baked product. Overall, the use of ‘ancient’ plant materials in making of sourdough bread seems to be a promising method for delivering a ‘clean labelling’ feature in bakery items, satisfying the consumer’s demand for healthy and naturally produced breads, as well as exhibiting quality attributes comparable to breads made from conventional wheat flours.

## Figures and Tables

**Table 1:** Bread formulations.

Sample Symbol	Flour Formulation	Sourdough (% *w*/*w* Flour Basis)
Control	Einkorn mixture ^1^ (100%)	-
Einkorn	Einkorn mixture (100%)	20
Einkorn + Spelt	Mixture of einkorn mixture (80%) with spelt (20%)	20
Einkorn + Acorn	Mixture of einkorn mixture (80%) with acorn (20%)	20
Einkorn + Lentil	Mixture of einkorn mixture (80%) with lentil (20%)	20
Einkorn + Barley	Mixture of einkorn mixture (80%) with barley (20%)	20
Einkorn + Poppy seed	Mixture of einkorn mixture (80%) with poppy seed (20%)	20
Einkorn + Linseed	Mixture of einkorn mixture (80%) with linseed (20%)	20
Spelt	Commercial spelt (100%)	20
Barley	Commercial barley (100%)	20
Common wheat	Commercial common wheat (*Triticum aestivum*) (100%)	20

^1^ Mixture of the 9 flour samples ground by the three different stones (prehistoric grinding tool replicas) and three grinding ways mixed in equal amounts.

**Table 2:** Starch gelatinization parameters of slurries (35% *w*/*w*) of the flour samples used for breadmaking; heating rate 5 °C/min.

Samples ^1^	Τ_ο_ (°C) ^2^	T_m_ (°C) ^2^	ΔΗ ^2^ (mJ/mg of Flour)
Barley	58.91 (±0.06) c ^3^	65.70 (±0.21) a	3.22 (±0.09) b
Common wheat	57.56 (±0.79) b	61.61 (±1.66) a	5.55 (±0.35) c
Einkorn	59.27 (±0.08) c	64.29 (±0.14) a	4.27 (±1.67) bc
Einkorn + Lentil	58.97 (±0.72) c	65.56 (±0.11) a	5.60 (±0.57) c
Lentil	64.74 (±0.24) d	73.98 (±0.39) b	0.93 (±0.46) a
Einkorn + Spelt	59.14 (±0.17) c	64.86 (±0.52) a	5.54 (±0.93) c
Spelt	56.15 (±0.26) a	63.42 (±1.51) a	4.55 (±0.42) bc
Einkorn + Barley	59.44 (±0.16) c	65.10 (±1.00) a	4.57 (±0.59) bc

^1^ Notation of samples is given in Table 1. ^2^ T_o_: onset starch gelatinization temperature, T_m_: peak starch gelatinization temperature and ΔH: apparent starch gelatinization enthalpy. ^3^ Values followed by the same letter for the same column are not significantly different (*p* > 0.05, Tukey’s test).
